# Case of Exomphalos, with Stricture and Other Diseases in the Abdomen

**Published:** 1818-07

**Authors:** Thomas Girdlestone


### *For the London Medical and Physical Journal.*

### *Case of Exomphalos, with Stricture and other Diseases in the Abdomen;* by THOMAS GIRDLESTONE, M.D.

AS the pages of the London Medical and Physical Journal were, during the years 1801 and 1802, much occupied by a controversy between Mr. Crowfoot and Dr. Longslow, and many other medical men, on Apoplexy, in consequence of a lady being attacked with dyspeptic syncope, which took place after dinner, and was greatly relieved by the constitution spontaneously effecting the expulsion of the contents of the stomach; the following sketch of the symptoms which preceded the death of this patient, with the appearances on the dissection, may prove interesting to many of your readers.

She remained, about ten years after the supposed apoplexy, in her usual health. She had, for twenty-four years before her death, laboured under a small exomphalos, for which she had been directed by the late Dr. Denman to keep her bowels in a soluble state, with senna and soluble tartar. About two years before her death, she complained of an intolerable pain on the left side in getting into her carriage. So severe was this pain, that she could not help screaming out: but, when once she was seated, the motion of the carriage gave her no uneasiness. For this pain she went up to town and consulted Dr. Pemberton. She was advised to keep her bowels open, as she had been before directed; and, after remaining some weeks in London, she returned without having experienced any great abatement of her symptom. In May 1813, I visited her; and, on finding that she had fever, which put on the appearance of the intermittents of that season, I apprehended that this was a fever of that description, till the next morning, when I had an opportunity of ascertaining the seat of the pain. Upon examining the part, I found, in the region of the kidney, a very large tumour, which was evidently the cause of all her uneasy sensations, and the fever that then existed. Upon enquiry, I found that neither Dr. Denman nor Dr. Pemberton had ever been acquainted with this tumour, since the lady had never been ill enough to keep her bed during their attendance on her. The history which I received from her was that twenty-four years before, when she was in a pregnant state, one of her children was very near falling, and, on the attempt to save him from falling, she gave herself considerable pain in that part, which was followed with the exomphalos, and a small swelling on the left side, which she never showed to any other person than to the late Dr. Manning of Norwich; who, at that time, thought it a mere steatomatous tumour, and of no consequence. About three weeks before I saw her, she began to think the exomphalos was the cause of the pain which she experienced on getting into a carriage, and therefore Mr. Martineau, of Norwich, had visited her. He, on examining the exomphalos, found that of no consequence; but he observed the tumour, which I have described, in the region of the kidneys. He and I frequently afterwards saw her together: and, as from the attitude in which she at different times laid it was difficult to determine whether an enlargement of the spleen or of the kidney occasioned the tumour, but as so much pain had always been experienced on the lumbar muscles being put into action on her entering into the carriage, I was induced to consider the kidney, rather than the spleen, enlarged. The tongue, very soon after the febrile appearance, put on that dark typhoid colour which is so common to old people, when some link of the chain necessary to life is giving way. Her sickness was constant; and, as she could retain no medicine but in the shape of a pill, small doses of calomel and opium were persisted in till the mucous glands of the tongue put on a better appearance; and injections about this time brought away an immense quantity of hardened fæces, which appeared, from the colour, hardness, and quantity, to have been long retained. As no medicine could be borne to purge the bowels, the injections were daily repeated, till nothing but mucus returned after each injection. This patient outlived the typhöid colour of the tongue a great many weeks. She had frequent inflations of the abdomen, with loss of power of the left side, which never returned to the lower extremities. The inflation of the abdomen was generally preceded by a temporary suspension of urine, and subsided on a return of a copious discharge of urine. The nates ulcerated from long lying, and the impossibility of making use of any other conveniency, with so hoary a subject, than folds of sheets to collect the contents of the bladder; and still, by the constant attention of many nurses, and great surgical attention, the ulcerated parts healed long before her death, which took place on the 28th of September, 1813.

At my particular request, an inspection of the thoracic and abdominal viscera was made the next day, by Mr. Hinchman Crowfoot, (as it was distant from Norwich for Mr. Martineau to attend,) in the presence of Dr. Wilson,

Mr. Primrose, Mr. Sutherland, Mr. William Crowfoot, and myself. The following were the appearances:—

The rupture at the navel contained only a small portion of omentum, which was healthy. The stomach and liver were sound. The gall-bladder was distended with bile and seventy-six gall-stones, which were not adhering to the gall-bladder, and all of a dark green colour, and of a uniform size. All the small intestines were sound. The cœcum was sound, but contained hardened balls of motion. The colon, to the left extremity of its arch, was filled with hardened fæces. At that part extended a stricture of a scirrhous nature, which had occasioned such obstruction as had induced sphacelation, rupture, and the escape of fæces into the cavity, so as to surround the kidney. This part of the colon, by adhering to the spleen, pancreas, and left kidney, in consequence of the pressure from the quantity of hardened fæces which it contained, had induced a cartilaginous appearance on the surface of those glands, and a diminution of the size of the pancreas and spleen, but not of the kidney, which, though surrounded with fæces, was perfectly sound. The colon, from this stricture to within two inches of the rectum, was nearly obliterated; and, though there was no stricture about the sphincter ani or rectum, yet the rectum contained a solid ball of fæces weighing sixteen ounces and a half. Excepting a considerable adhesion of the left pleura pulmonalis to the pleura costalis, the thoracic viscera were sound. And, as no symptoms about the head existed during her illness to denote any disease in the brain, no examination of that part was thought necessary.

*Yarmouth; June* 9, 1818.

---

*For the London Medical and Physical Journal.*

